# EZHIP is a specific diagnostic biomarker for posterior fossa ependymomas, group PFA and diffuse midline gliomas H3-WT with EZHIP overexpression

**DOI:** 10.1186/s40478-020-01056-8

**Published:** 2020-11-05

**Authors:** C. Antin, A. Tauziède-Espariat, M.-A. Debily, D. Castel, J. Grill, M. Pagès, O. Ayrault, F. Chrétien, A. Gareton, F. Andreiuolo, E. Lechapt, P. Varlet

**Affiliations:** 1 Department of Neuropathology, GHU Paris-Neurosciences, Sainte-Anne Hospital, 1, rue Cabanis, 75014 Paris, France; 2 UMR8203, Vectorologie et thérapeutiques anticancéreuses, CNRS, Gustave Roussy, Univ. Paris-Sud, Univ. Paris-Saclay, 94805 Villejuif Cedex, France; 3 Univ. Evry, Université Paris-Saclay, 91057 Evry Cedex, France; 4 Department of Pediatric Oncology, Gustave Roussy Institute, Univ. Paris-Sud, Universite Paris-Saclay, 94805 Villejuif, France; 5 CNRS UMR, INSERM, Institut Curie, PSL Research University, 91898 Orsay, France; 6 CNRS UMR 3347, INSERM U1021, Université Paris Sud, Université Paris-Saclay, 91898 Orsay, France

In the central nervous system (CNS), the loss of H3K27me3 expression constitutes the hallmark of two different tumor types: diffuse midline glioma (DMG), H3K27-mutant and posterior fossa ependymoma, group PFA (PFA-EPN). In the former, mutations in histone genes (mostly *H3F3A* K27M and *HIST1H3B* K27M), present in about 97% of DMG, inhibit the activity of the Polycomb Repressive Complex 2 (PRC2) methyltransferase [1]. However, these mutations are rare in PFA-EPN (accounting for ≈ 4% of cases) [2]. Recent molecular advances have shown that the Enhancer of Zest Homologs Inhibitory Protein (EZHIP) is overexpressed (due to gene overexpression rather than mutations of the *CXorf67* gene) in the large majority of PFA-EPN, and in the remaining cases of DMG showing H3K27me3 loss but lacking histone gene (H3) mutations [1–3]. Indeed, this overexpression mimics the mechanism of histone gene mutations on PRC2 [4]. Usually, the current routine immunohistochemical (IHC) panel in pediatric neuropathology includes H3K27me3 and H3K27M antibodies but not EZHIP. The aim of our study was to evaluate the sensitivity and specificity of the EZHIP biomarker in a large cohort of pediatric tumors, including the most common tumor types, which arise in the brainstem and the posterior fossa.

We performed IHC for EZHIP using the CXorf67 antibody (Polyclonal; 1:75 dilution; Sigma-Aldrich; Bromma, Sweden) on 3 µm-thick sections of formalin-fixed, paraffin-embedded tissue samples of these tumors, performed on an Omnis automate. Our study included a total of 311 cases: 298 pediatric tumors of different subtypes (gliomas, embryonal, and ependymal tumors with a morphomolecular diagnosis including DNA-methylation profiling), and 13 posterior fossa ependymomas, Group PFB (for details see Table 1). This series includes some of the tumors previously reported [1]. The IHC were performed on whole sections in 266 cases and on a TMA (tissue microarray) of 45 ependymomas as a validation cohort which included PFA (n = 37), H3K27-mutant (n = 2) and PFB (n = 6). The IHC stainings were scored by three neuropathologists (ATE, PV and EL) independently.

**Table 1:** Immunohistochemical results of EZHIP in our series

Tumor types	EZHIP [n (%)]
Diffuse astrocytic and oligodendroglial tumors	
Astrocytoma, *IDH*-mutant, grade 2	0/2 (0)
Oligodendroglioma, *IDH*-mutant and 1p19q codeleted, grade 2	0/3 (0)
Epithelioid glioblastoma	0/1 (0)
Astrocytoma, *IDH*-mutant, grade 4	0/3 (0)
DMG, H3K27-mutant	0/24 (0)
DMG, H3K27-WT with EZHIP overexpression	13/13 (100)
HGG with MSI	
CMMRD	0/6 (0)
Lynch syndrome	0/4 (0)
Diffuse glioma, H3.3 G34-mutant	0/10 (0)
HGG, *MYCN*-amplified	0/9 (0)
Glioblastoma, *IDH*-WT	0/10 (0)
Other astrocytic tumors	
Pilocytic astrocytoma	0/10 (0)
High-grade astrocytoma with piloid features	0/1 (0)
Pleomorphic xanthoastrocytoma with anaplastic features	0/9 (0)
Ependymal tumors	
Myxopapillary ependymoma	0/6 (0)
Posterior fossa ependymoma	
Group PFA	47/47 (100)
Group PFA, H3K27-mutant	0/2 (0)
Group PFB	0/19 (0)
Supratentorial EPN	
*YAP1*-fusion-positive	0/5 (0)
*C11orf95* fusion-positive	0/17 (0)
Subependymoma	0/2 (0)
Neuronal and mixed neuronal-glial tumors	
Diffuse leptomeningeal glioneuronal tumor	0/1 (0)
Tumors of the pineal region	
Pineoblastoma	0/10 (0)
Embryonal tumors	
Medulloblastoma, group 3	0/5 (0)
Medulloblastoma, group 4	0/5 (0)
Medulloblastoma, SHH-activated	0/10 (0)
Medulloblastoma, WNT-activated	1/10 (10)^a^
Embryonal tumors with multilayered rosettes, *C19MC*-amplified	0/10 (0)
AT/RT	
AT/RT MYC	1/3 (33)^a^
AT/RT SHH	0/4 (0)
AT/RT TYR	0/3 (0)
CNS tumor with *BCOR* internal tandem duplication	0/8 (0)
CNS high-grade neuroepithelial tumor with *MN1* alteration	0/8 (0)
Germ cell tumors	
Germinoma	29/31 (94)

*AT/RT* atypical teratoid/rhabdoid tumor, *CNS* central nervous system, *EPN* ependymoma, *HGG* high-grade glioma, *MSI* microsatellite instability, *WT* wildtype

^a^1 case presents a focal expression of EZHIP (< 1% of tumor cells)

The IHC results (including the validation cohort) are detailed in Table 1. A strong and diffuse EZHIP nuclear staining (> 90% of immunopositive tumor cells) was observed in all DMG, H3-wildtype with EZHIP overexpression (n = 13) (Fig. 1A–C) and all PFA-EPN (n = 47) (Fig. 2A–C and Additional file 1: Figure S1), except the two EPN, H3K27-mutants (Fig. 2G–I). The majority of germinomas exhibited a strong nuclear immunostaining (94%, 29/31 cases) associated with a loss of H3K27me3 trimethylation (Fig. 1G–I and Additional file 2: Figure S2). In all other diagnoses, tumor cells were immunonegative except for two cases: one atypical teratoid/rhabdoid tumor (AT/RT) belonging to the *MYC* methylation class and one medulloblastoma, WNT-activated. These two cases exhibited only focal expression (< 1% of immunopositive tumor cells) (data not shown). This low protein expression of EZHIP was correlated with a normal level of *CXorf67* gene expression at the mRNA level. Thus, the specificity and the sensitivity of the IHC were evaluated as 99% and 98% respectively.

**Fig. 1:**
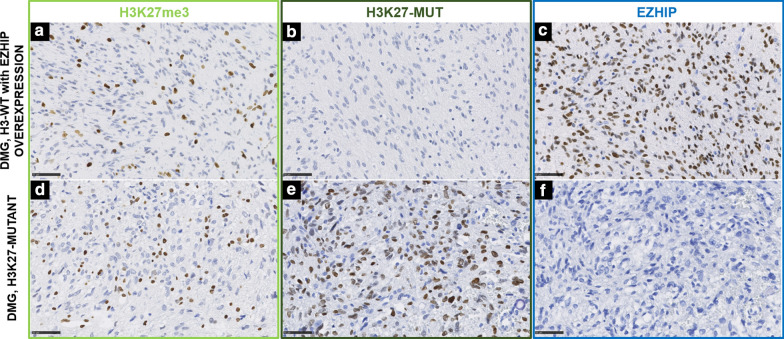
EZHIP expression in diffuse midline gliomas. A distinct H3K27me3 loss (**A**, magnification, ×400) in one case of diffuse midline glioma with EZHIP overexpression, without expression of H3K27-mutant protein (**B**, magnification, ×400) and with strong positive EZHIP expression (**C**, magnification, ×400). A case of diffuse midline glioma, H3K27-mutant with a loss of expression of H3K27me3 (**D**, magnification, ×400), nuclear expression of H3K27-mutant protein (**E**, magnification, ×400), and without expression of EZHIP (**F**, magnification, ×400). *DMG* diffuse midline glioma, *WT* wildtype. Black scale bars represent 50 μm

**Fig. 2:**
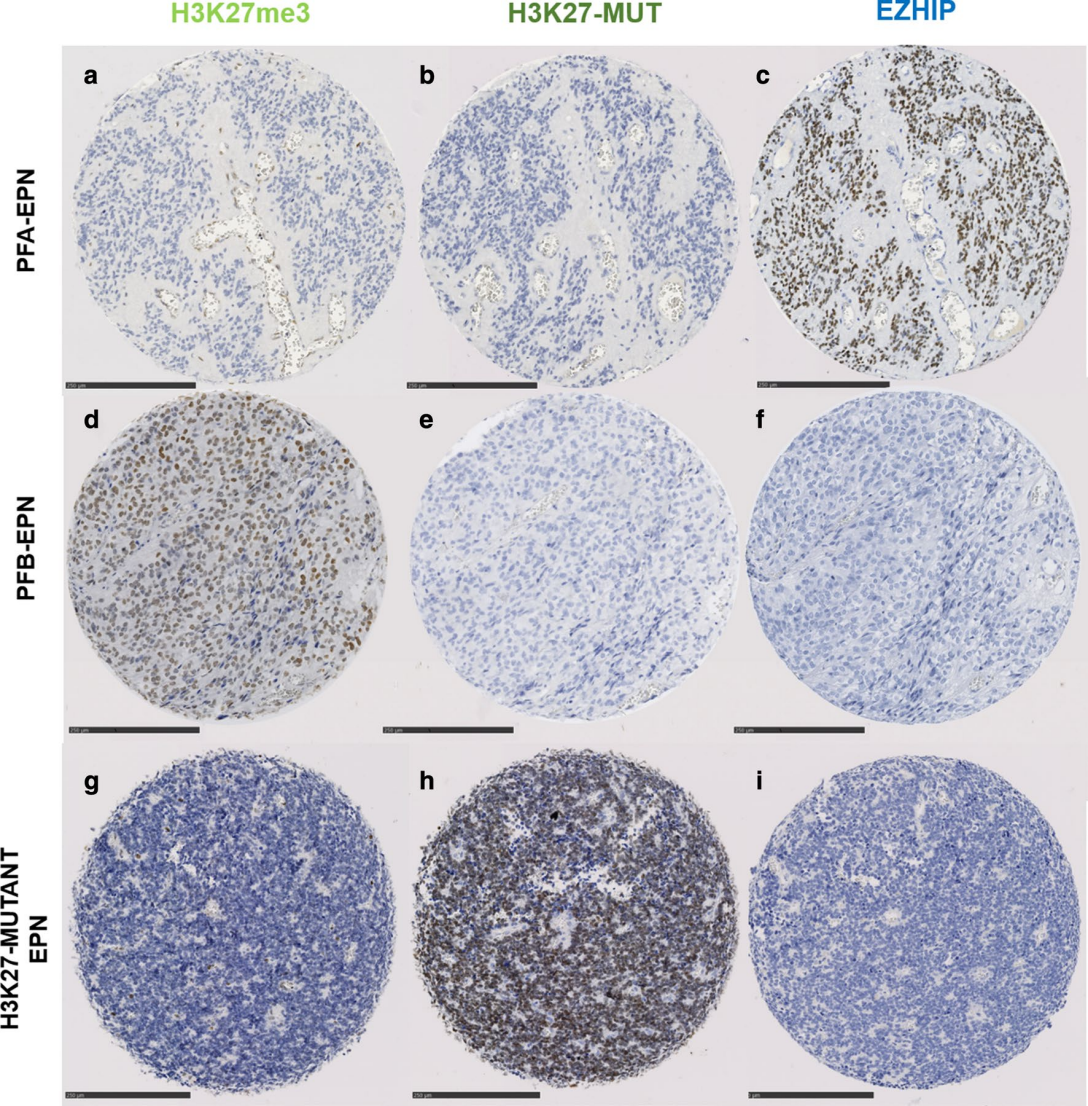
EZHIP expression in ependymomas. The first line shows immunohistochemical analyses of a case of PFA-EPN exhibiting a loss of H3K27me3 (**A**, magnification, ×170), no H3K27-mutant protein (**B**, magnification, ×170) and an EZHIP overexpression with a strong and diffuse nuclear staining (**C**, magnification, ×170). The second line represents a case of PFB-EPN with, as expected, no loss of H3K27me3 expression (**D**, magnification, ×170), no H3K27-mutant protein expression (**E**, magnification, ×170), and negative EZHIP immunostaining (**F**, magnification, ×170). The last case (line 3) corresponds to a variant of PFA-EPN with H3K27-mutation exhibiting an H3K27me3 loss (**G**, magnification, ×170), a strong positive staining for H3K27-mutant protein (**H**, magnification, ×170) and no EZHIP expression (**I**, magnification, ×170). Black scale bars represent 250 μm

This work constitutes the first study of the sensitivity/specificity of EZHIP immunoexpression in a large cohort of CNS tumors. Our results highlighted that nuclear EZHIP expression must be diffuse and strong to be interpreted as overexpressed. Thus, EZHIP IHC constitutes a fast, low-cost and conservative tissue-consuming method to detect *CXorf67* overexpression, suitable for small samples (particularly in brainstem biopsies), but also in samples that contain few tumor cells. The IHC may also help to evaluate the quality of resection (surgical limits). Indeed, a nuclear immunopositivity is easier to interpret than the loss of H3K27me3. Our work highlighted the robust specificity of EZHIP staining in all PF ependymomas, group PFA and in all DMG, H3-wildtype with EZHIP overexpression, ruling out the main differential diagnoses encountered in children in the brainstem and in the posterior fossa (Table 1). All germinomas except two exhibited a strong positivity for EZHIP concomitant with a loss of H3K27me3 as published previously [2, 7]. Concerning HGG, *MYCN*-amplified none of our 9 cases (confirmed by DNA-methylation profiling and previously reported [5, 6]), were immunopositive, contrarily to a previous study which reported an expression of EZHIP in 13% of cases [1]. Moreover, this biomarker may represent a diagnostic but also a prognostic tool. Indeed, PFA-EPN were associated with a poorer prognosis than PFB-EPN, and patients with DMG overexpressing EZHIP presented a better overall survival compared to DMG, H3K27-mutant [1].

To conclude, we demonstrated that EZHIP IHC is a highly specific and sensitive biomarker for identifying PFA-EPN and DMG, H3-wildtype, with EZHIP overexpression, and should be part of the neuropathologist’s routine panel of antibodies.

## Supplementary information

Additional file 1: Figure S1. EZHIP expression in ependymomas of different grades. The first line shows immunohistochemical analyses of a case of grade 2 PFA-EPN (**A**, HPS magnification, 400x) exhibiting a loss of H3K27me3 (**B**, magnification, 400x), and an EZHIP overexpression with a strong and diffuse nuclear staining (**C**, magnification, 400x). The second line represents a case of grade 3 PFA-EPN with microvascular proliferation and mitoses (white arrowheads) (**D**, HPS magnification, 400x), with a loss of H3K27me3 expression (**E**, magnification, 400x), and strong and diffuse EZHIP immunopositivity (**F**, magnification, 400x). HPS: Hematoxylin Phloxin Saffron. Black scale bars represent 50 μm.

Additional file 2: Figure S2. EZHIP expression in germinomas. A case of germinoma with H3K27me3 loss (**A**, magnification, 400x), no expression of H3K27-mutant protein (**B**, magnification, 400x), and strong and diffuse nuclear immunoexpression of EZHIP (**C**, magnification, 400x). Black scale bars represent 50 μm.
