# Supplementary figures and images for: EZHIP is a specific diagnostic biomarker for posterior fossa ependymomas, group PFA and diffuse midline gliomas H3-WT with EZHIP overexpression
Source: Acta Neuropathol Commun. 2020 Nov 5;8:183. doi: 10.1186/s40478-020-01056-8 (PMC7643397; doi:10.1186/s40478-020-01056-8)

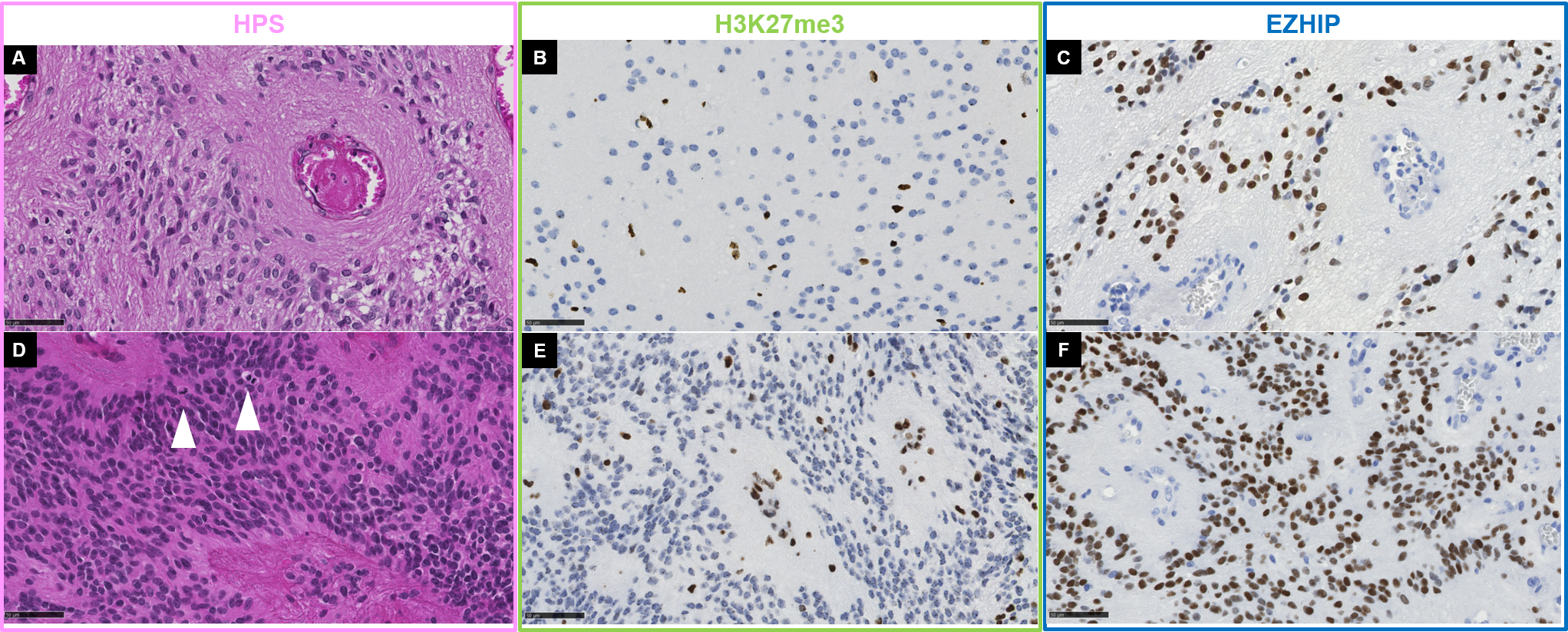

Supplement: Supplementary file 1 — EZHIP expression in ependymomas of different grades. The first line shows immunohistochemical analyses of a case of grade 2 PFA-EPN (A, HPS magnification, 400x) exhibiting a loss of H3K27me3 (B, magnification, 400x), and an EZHIP overexpression with a strong and diffuse nuclear staining (C, magnification, 400x). The second line represents a case of grade 3 PFA-EPN with microvascular proliferation and mitoses (white arrowheads) (D, HPS magnification, 400x), with a loss of H3K27me3 expression (E, magnification, 400x), and strong and diffuse EZHIP immunopositivity (F, magnification, 400x). HPS: Hematoxylin Phloxin Saffron. Black scale bars represent 50 μm. [file 40478_2020_1056_MOESM1_ESM.tif]

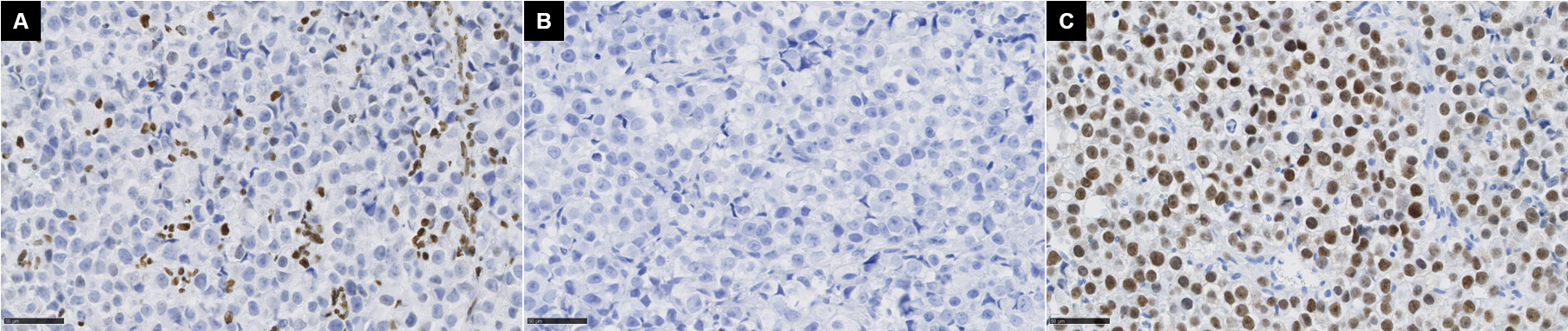

Supplement: Supplementary file 2 — EZHIP expression in germinomas. A case of germinoma with H3K27me3 loss (A, magnification, 400x), no expression of H3K27-mutant protein (B, magnification, 400x), and strong and diffuse nuclear immunoexpression of EZHIP (C, magnification, 400x). Black scale bars represent 50 μm. [file 40478_2020_1056_MOESM2_ESM.tif]
